# Surveillance of SARS-CoV-2 Genetic Variants in the Polish Armed Forces Using Whole Genome Sequencing Analysis

**DOI:** 10.3390/ijms241914851

**Published:** 2023-10-03

**Authors:** Katarzyna Skuza, Pawel Rutyna, Lukasz Krzowski, Lukasz Rabalski, Tomasz Lepionka

**Affiliations:** 1 Biological Threats Identification and Countermeasure Center, General Karol Kaczkowski Military Institute of Hygiene and Epidemiology, Lubelska 4, 24-100 Pulawy, Poland; katarzyna.skuza@wihe.pl; 2 Chair and Department of Medical Microbiology, Medical University of Lublin, 1 Chodzki, 20-093 Lublin, Poland; pawel.rutyna@umlub.pl; 3 Biomedical Engineering Centre, Institute of Optoeletronics. Military University of Technology, 2 Gen. Sylwestra Kaliskiego, 00-908 Warsaw, Poland; lukasz.krzowski@wat.edu.pl; 4 Department of Recombinant Vaccines, Intercollegiate Faculty of Biotechnology University of Gdansk and Medical University of Gdansk, Abrahama 58, 80-307 Gdansk, Poland

**Keywords:** SARS-CoV-2, COVID-19, outbreak, military, variants, genomic epidemiology

## Abstract

Military operations involve the global movement of personnel and equipment, increasing the risk of spreading infectious pathogens such as SARS-CoV-2. Given the continuous engagement of the Polish Armed Forces in overseas operations, an active surveillance program targeting Variants of Concern (VOC) of SARS-CoV-2 was implemented among military personnel. Screening using RT-qPCR tests was conducted on 1699 soldiers between November 2021 and May 2022. Of these, 84 SARS-CoV-2 positive samples met the criteria for whole genome sequencing analysis and variant identification. Whole genome sequencing was performed using two advanced next-generation sequencing (NGS) technologies: sequencing by synthesis and nanopore sequencing. Our analysis revealed eleven SARS-CoV-2 lineages belonging to 21K, 21L, and 21J. The predominant lineage was BA.1.1 (57% of the samples), followed by BA.1 (23%) and BA.2 (6%). Notably, all identified lineages detected in post-deployment screening tests were classified as VOC and were already present in Poland, showing the effectiveness of the Military Sanitary Inspection measures in mitigating the COVID-19 spread. Pre-departure and post-mission screening and isolation successfully prevented SARS-CoV-2 VOC exportation and importation. Proactive measures are vital in minimizing the impact of COVID-19 in military settings, emphasizing the need for continued vigilance and response strategies.

## 1. Introduction

Throughout history, war epidemics have posed significant threats to the combat capabilities of armies, often resulting in the suspension or abandonment of military operations and severe consequences for civilian populations [1]. However, advancements in military hygiene, disease control methods, and medical interventions, including vaccination, chemoprophylaxis, antibiotics, medical treatment, personal protection, and vector control measures, have led to a decline in the incidence of infectious diseases among both soldiers and civilians since the Russo-Japanese War and World War I [2].

Poland, as an active participant in international relations and committed to strengthening international peace and security, has been actively engaged in international missions since 1953. Over the years, more than 120,000 soldiers and military personnel have participated in over 89 operations. The presence of Polish Armed Forces (PAF) in 12 Military Contingents across various continents exposes them to endemic infectious diseases [3].

In December 2019, the world encountered a novel challenge in the form of an outbreak of pneumonia caused by a newly emerging pathogen, severe acute respiratory syndrome coronavirus 2 (SARS-CoV-2). This viral agent demonstrated epidemic potential and quickly spread, leading to a global pandemic known as coronavirus disease (COVID-19) [4,5]. COVID-19 affects multiple organs and can result in long-term effects, often referred to as long COVID-19, in the human body [6]. While the main symptoms of COVID-19 include cough, runny/stuffy nose, fatigue/lethargy, sore throat, headache, and fever, systematic reviews have revealed that up to 40% of cases are asymptomatic, with asymptomatic individuals playing a significant role in the transmission of the disease [7].

The rapid spread of COVID-19, coupled with changes in the pathogenicity of SARS-CoV-2, highlights its ability to evolve continuously through genetic mutations or viral recombination during replication [8]. This was further evidenced by the emergence of a new SARS-CoV-2 variant, B.1.1.7, in Southern England in September 2020. Clinically, this variant demonstrated a higher transmission rate, increased severity of illness, and potential vaccine escape capability [9]. In response, the European Centre for Disease Prevention and Control (ECDC) recommended surveillance of SARS-CoV-2 Variants of Concern (VOC) among member states through virus genome sequencing. In 2022, a new classification system for variants was introduced, comprising Variant Being Monitored (VBM), Variant of Interest (VOI), Variant of Concern (VOC), and Variant of High Consequence (VOHC) [10,11,12].

Since the onset of the pandemic, the Polish Armed Forces have actively contributed to mitigating the epidemic and its consequences through various measures. Consequently, similar to healthcare workers, military personnel have been exposed to COVID-19 infections. Moreover, their engagement in military missions and operations in countries with high infection rates has increased the risk of VOC transmission among military personnel. As a result, the Department of the Military Medical Service Ministry of National Defence implemented a 14-day quarantine for all troops deployed overseas, along with pre-and post-deployment testing for SARS-CoV-2. These measures were also extended to NATO alliance troops deployed or redeployed from Poland [13].

To facilitate the identification of SARS-CoV-2 infections among military personnel, the Military Sanitary Inspection established a network of 14 military medical diagnostic laboratories. Additionally, the Biological Threats Identification and Countermeasure Centre of the Military Institute of Hygiene and Epidemiology was assigned the task of VOC surveillance based on recommendations from the Military Sanitary Inspection. Specific focus was given to the following cases:

- Individuals returning from deployments outside the country as part of the Polish Military Contingent, international exercises, or service in Polish institutions;
- Convalescent individuals after previous COVID-19 infection (reinfection) occurring more than 60 days after the last positive diagnostic test for SARS-CoV-2 (using molecular RT-PCR or rapid antigen test);
- Individuals vaccinated with two doses of Pfizer-BioNTech/Moderna/AstraZeneca or one dose of Johnson and Johnson vaccine, with recurrence occurring at least 14 days after the final dose;
- Infected individuals exhibiting atypical clinical symptoms of COVID-19 with rapid infection dynamics;
- 20% of other samples tested positive for SARS-CoV-2 RNA using the RT-PCR method.

The objective of genomic surveillance for VOC was to prevent the transmission of new VOCs to Poland or Polish Armed Forces deployment sites.

This manuscript presents the results of active surveillance for VOCs of SARS-CoV-2 conducted between November 2021 and May 2022, emphasizing its epidemiological importance for soldiers and military personnel [10].

## 2. Results and Discussion

**Figure 1:**
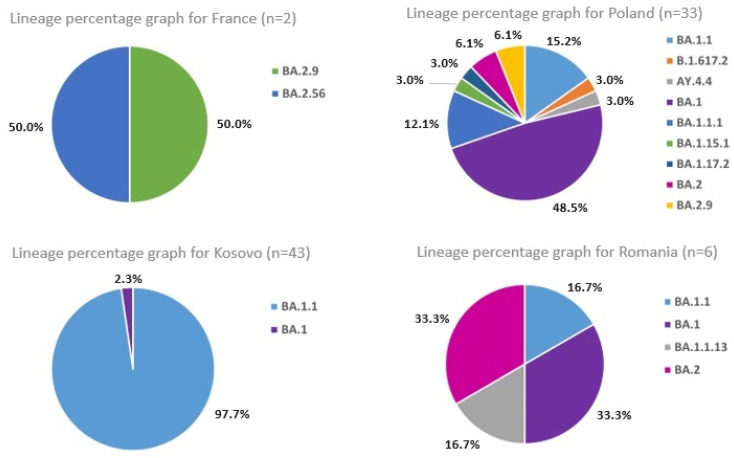
Distribution of identified SARS-CoV-2 lineages in samples from various locations. The figure illustrates the share of each lineage in the sample pool, highlighting the prevalence of certain lineages and their potential implications for the transmission and severity of the disease. ‘n’ represents the number of samples from each examined location.

The analysis of viral genomes aimed to identify possible genetic variants of the coronavirus and track the virus’s transmission and spread among soldiers serving in the country and deployed overseas. Of 223 SARS-CoV-2 positive samples, 84 met the criteria for variant identification, according to the European Centre for Disease Prevention and Control (ECDC) [12]. Variant identification was performed using the Pangolin tools for lineage identification [13]. Instead of tracking individual mutations, the focus was on Pango lineages, which represent groups or clusters of infections with shared ancestry and have epidemiological significance [14]. Clade assignments were also determined based on Nextstrain classifications [15]. The detailed data on the sequenced samples and their vaccination status can be found in supplementary Table S1, and the distribution of identified lineages for each country is presented in Figure 1.

The analysis revealed significant genetic variation within the examined samples, with 11 lineages and sublineages identified. The most frequent Pango lineage was BA.1.1, observed in 57% of the samples, followed by BA.1 and BA.2 (23% and 6% of the sample pool, respectively). According to Nextstrain clades, the most common variant was 21K, found in 88% of the samples, followed by 21L and 21J (10% and 2% of the samples, respectively).

**Figure 2:**
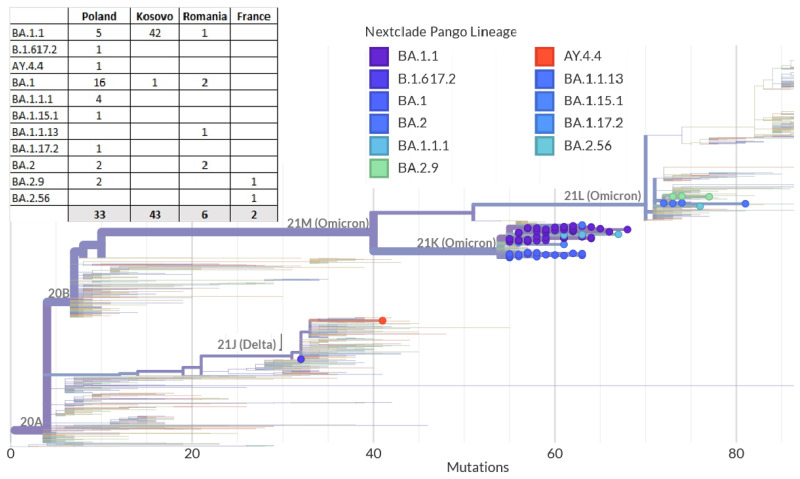
Rectangular phylogenetic tree of the SARS-CoV-2 lineages. Coloured dots stand for samples assigned to the corresponding Pango Lineage [15,16].

The sequences were mapped on a phylogenetic tree of SARS-CoV-2 genomes using the Nextstrain platform, which visualizes the nucleotide and amino acid differences between the genomes (Figure 2). Additionally, a phylogenetic tree was generated using the IQ-TREE method for multiple alignments of whole genomes of SARS-CoV-2 (Figure S2 in Supplementary Materials). The figure presents the tree with the timeline combined with information regarding the identified WHO clade and the place of origin of the sample. The green clade stands for Delta strains, blue—Omicron (21K) strains, and yellow—Omicron (21L). The red dots in the timeline represent strains from Poland, green from Kosovo, blue from Romania, and yellow from France.

Using the AudacityInstant tool (v5.1.0) implemented in the GISAID EpiFlu™ Database, we analysed the phylogeography of all SARS-CoV-2 isolates described in this publication. This tool searches the entire EpiCoV database to find the most similar virus sequences (differing by 0–3 nucleotides across the entire genome length). Among the samples obtained from soldiers pre-deployment, we identified four groups of related sequences, totalling nine sequences. The remaining twenty-four sequences do not indicate close relationship among themselves. Notably, only for the sequence EPI_ISL_11313689 (a variant widely spread in Europe) was a similar sequence found in Kosovo three months later. The remaining sequences from this group (obtained from soldiers pre-mission) were not found in the countries to which the personnel were assigned to go. Among the sequences obtained from soldiers during isolation post-missions, we can distinguish seven groups with a total of thirty-nine sequences (all from soldiers returning from Kosovo) and eleven isolates without connection to other samples. Analysing individual groups, we noticed that similar sequences were not detected in Poland after the soldiers’ arrival from the mission. The sequence EPI_ISL_12253980, which represents a genotype group numerous in Kosovo but not found in Poland after the mission, is noteworthy. Sequences representing other genotypes such as EPI_ISL_10008059, EPI_ISL_10008060, EPI_ISL_10008062, EPI_ISL_11349026, EPI_ISL_11349033, and EPI_ISL_11313687 formed only single clusters composed of isolates from this study but were very numerous in other countries such as Germany, the UK, Turkey, or Israel. Additionally, the sequence EPI_ISL_11349026 belongs to a group very widespread in southern Europe among countries bordering Kosovo but did not occur in Poland except for nine cases among soldiers. Among all analysed sequences, not a single one was found in the soldiers’ base region and then found in Poland. 

**Figure 3:**
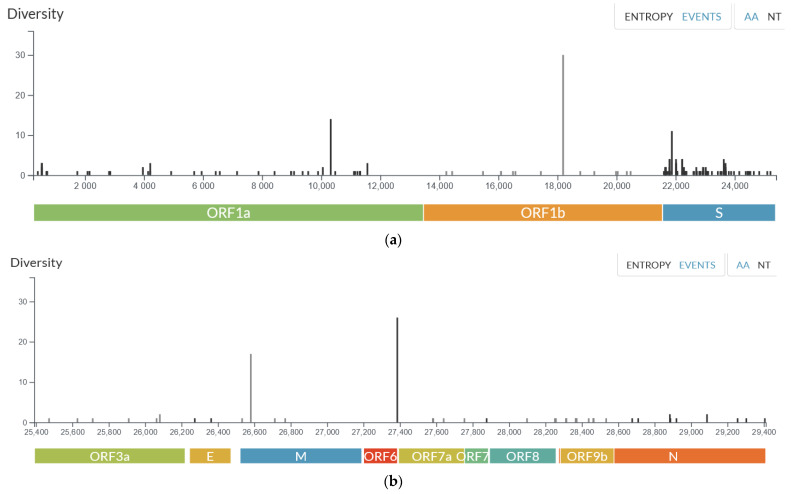
(**a**). Frequency of identified mutations across reference SARS-CoV-2 genes from *ORF1a* to gene *S* [16]. (**b**). Frequency of identified mutations across reference SARS-CoV-2 genes from *ORF3a* to gene *N* [16].

The frequency of identified amino acid mutations in the samples compared to the reference genome was also analysed (Figure 3a,b). The highest number of mutations was observed in the S protein, with the most frequent mutations identified in *ORF1b* (P314L in all samples), the M protein (D3G and A63T in more than 85% of samples), *ORF1a* (T3255I and P3395H in more than 97% of samples), and the S protein (T478K, D614G in all samples). The P314L mutation refers to a change in the amino acid at position 314 in the *ORF1b* (Open Reading Frame 1b) gene of the SARS-CoV-2 virus. This mutation has been observed in all the examined samples. *ORF1b* encodes non-structural proteins involved in viral replication and transcription. It is suggested that the functional significance of the P314L mutation in *ORF1b* may result in affecting *RdRp* activity and viral replication in SARS-CoV-2, as well as enhanced resistance to some antiviral drugs like remdesivir and favipiravir, as this mutation is located close to the drug binding region [17,18].

The M protein, also known as the membrane protein, Is involved in assembling the viral envelope. D3G and A63T mutations may play an important role in host–cell interaction [19]. The S protein, or Spike protein, is a crucial component of the SARS-CoV-2 virus that enables viral entry into host cells. The two mutations, T478K and D614G, identified in all samples, have been known to enhance the virus binding with the host ACE2 receptor, resulting in increasing viral infectivity and transmission rate and immune evasion via escape from antibody neutralization [20].

The genomic changes described above shed light on the most prevalent in the examined pool and certainly do not exhaust the knowledge regarding their potential impact on the characteristics of the virus. Also, as we mentioned above, in the pool of 83 genomes described in this work, we did not identify any private mutations that were preserved in the Polish population. The mutations listed in the following subsections are lineage-defining and have been widely described. Therefore, we do not discuss their exact properties and characteristics in this manuscript. 

The results showed that the examined soldiers were infected with a range of SARS-CoV-2 lineages. The following subsections discuss the features of the identified lineages in relation to the timeline and mission locations.

### 2.1. Delta (21J)—November 2021:

**Figure 4:**
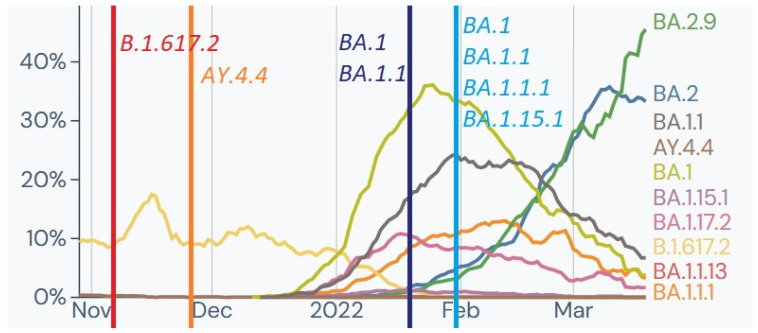
Tracked lineages from November 2021 to March 2022 in Poland [21]. *Y*-axis shows the percentage of lineage circulating in Poland; *X*-axis represents the timeline in months. Vertical coloured lines mark date of sampling with identified clades at the time.

Out of 79 soldiers tested for SARS-CoV-2 as a screening examination before deployment to an overseas mission, two tested positive and were selected for whole genome sequencing (WGS). The analysis identified two genomes belonging to clade 21J (Delta) and Pango lineages B.1.617.2 and AY.4.4. This clade, first detected in India, was a Variant of Concern (VOC) from 11 May 2021, to 11 June 2022 and was prevalent in Europe and Poland at the time of testing (Figure 4).

The two genomes carried characteristic Spike mutations (L452R and P681R) that impact antibody binding, and one sample had additional unique nucleotide substitutions (C21575T and G24348C) resulting in amino acid mutations (L5F and S929T). Other specific mutations were found in the N protein, *ORF1a*, *ORF1b*, and *ORF7b* [22,23,24].

### 2.2. Omicron 21K—January–February 2022

**Figure 5:**
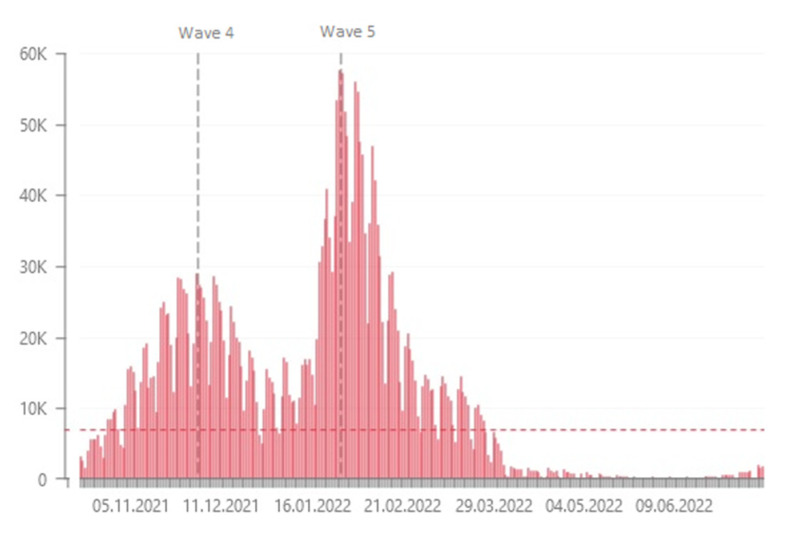
The daily number of SARS-CoV-2 infections in Poland [25]. *Y*-axis represents the number of cases; *X*-axis represents the timeline in months.

The period between January and February was unique in terms of the incidence of COVID-19 and was referred to as the fifth wave (Figure 5) [25]. During this phase, we identified 211 positive samples and selected 63 for WGS. Sixteen samples from a home outbreak belonged to Nextstrain clade 21K and Pango lineages B.1, B.1.1, and B.1.1.1. The Omicron variant (21K) had emerged in South Africa in November 2021 but had not been previously identified among Polish military personnel. The variant was considered a VOC due to significant mutations in the *S* gene affecting receptor binding and antibody recognition. All the genomes carried characteristic Spike mutations associated with increased transmissibility and immune escape. They also had a three-amino-acid deletion in *ORF1a* and Nucleocapsid mutations associated with increased viral loads [26,27,28].

Sixteen samples tentatively identified as a home outbreak belonged to Nextstrain clade 21K and three Pango lineages: B.1, B.1.1, and B.1.1.1. Despite the Omicron variant having arisen in November 2021 in South Africa, we identified it among polish military personnel in January 2022. Since samples were collected from the outbreaks inside the country, it was not connected with overseas missions. The 21K variant has been considered a VOC since 26 November 2021 to date, primarily due to the significant number of mutations in the *S* gene affecting the receptor binding domain and N-terminal domain, influencing the ACE2 binding and antibody recognition [26,27].

All the genomes carried characteristic Spike mutations such as H655Y, N679K, and P681H known as a cluster of mutation at the S1-S2 furin cleavage site associated with increased transmissibility; S:Q498R and S:N501Y increased the binding affinity to ACE2; and S:E484A has been associated with immune escape. Additionally, all the genomes were characterized by a three amino-acid deletion in *ORF1a*: L3674-, S3675-, G3676-, which was linked with the enhancement of an innate immune evasion as well as two Nucleocapsid mutations (R203K and G204R) associated with increased viral loads [28].

Further analysis of internal epidemiological outbreaks (EPI_ISL_9963996; EPI_ISL_10008058; EPI_ISL_9963994; EPI_ISL_12253987; EPI_ISL_12253985; EPI_ISL_9694168; EPI_ISL_9694169; EPI_ISL_12253986—see Supplementary Table S1) also confirmed the dominance of the Omicron 21K variant, except that one of the samples was identified as BA.1.15.1 (EPI_ISL_12253986) with a unique Spike mutation in S2 region (I1081V), two *ORF7a* (Q62- and F63H) mutations, and Nucleocapsid mutation D343G. Especially the last one may be of particular interest since it is linked with an increase in the ability of domain conformational rearrangement required for the dimerization processes [29].

**Figure 6:**
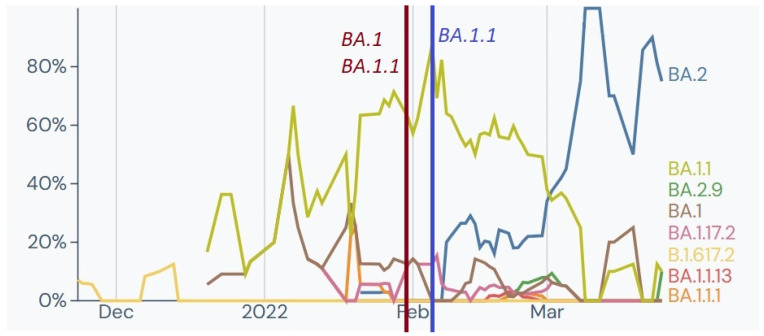
Tracked lineages from December 2021 to April 2022 in Kosovo [21]. *Y*-axis shows the percentage of circulating variants in Kosovo; *X*-axis represents the timeline in months. Vertical coloured lines mark date of sampling with identified clades at the time.

Additionally, 44 SARS-CoV-2 genomes from military personnel repatriated from the mission in Kosovo were analysed, and all belonged to Nextstrain clade 21K, predominantly Pango lineage BA.1.1. This indicated a cluster of infections with a short transmission chain. The observed lineages were prevalent in Kosovo at the time (Figure 6).

### 2.3. Omicron 21L

**Figure 7:**
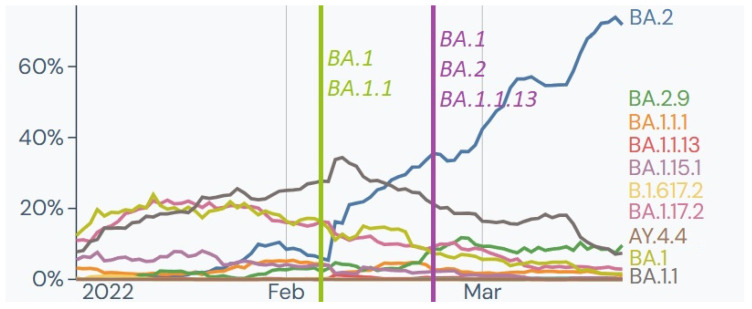
Tracked lineages from December 2021 to April 2022 in Romania [21]. *Y*-axis shows the percentage of lineage circulating in Romania; *X*-axis represents the timeline in months. Vertical coloured lines mark date of sampling Polish soldiers with identified clades at the time.

In February, 89 samples from soldiers deployed from Romania tested positive for SARS-CoV-2 infection, and six samples underwent WGS analysis. Samples collected in the first half of the month were identified as Nextstrain clade 21K and Pango lineages B.1, B.1.1, and BA.1.1.13. In the second half of February, two samples were identified as clade 21L and Pango lineage BA.2. These results were consistent with the variants and lineages observed in Romania, with BA.1, BA.1.1, and BA.2 being the most frequent (Figure 7). 

Four samples from a home outbreak in the first half of February were identified as 21L Omicron, with two belonging to BA.2 and two to BA.2.9 Pango lineages. These variants were less frequently observed then but became predominant the following month. The 21L variant carried additional Spike (S:T19I, S:V213G, S:S371F, S:T376A, S:D405N) and *ORF1a* (L3201F) mutations, indicating potential origins outside of Africa [30].

**Figure 8:**
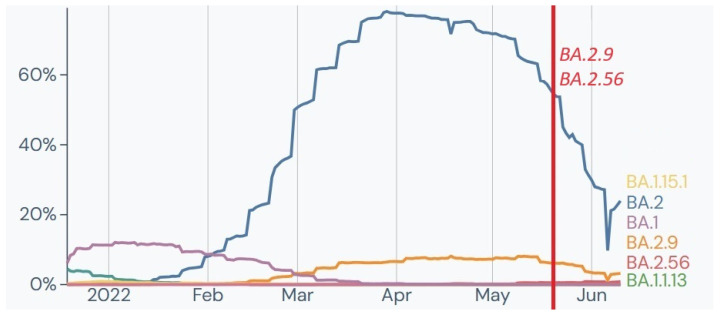
Tracked lineages from January 2022 to June 2022 in France [21]. *Y*-axis shows the percentage of lineage circulating in France; *X*-axis represents the timeline in months. Vertical coloured line marks date of sampling of Polish soldiers with identified clades at the time.

Also, two samples from soldiers returning from France in May were identified as 21L Omicron belonging to BA.2.9 and BA.2.56 Pango lineages (Figure 8). Interestingly, both genomes presented identical private mutations A4184G, which later in the literature was described as characteristic for XAG recombinant lineage, considered as recombination between BA.1 and BA.2 [31]. 

## 3. Materials and Methods

### 3.1. Study Design

**Figure 9:**
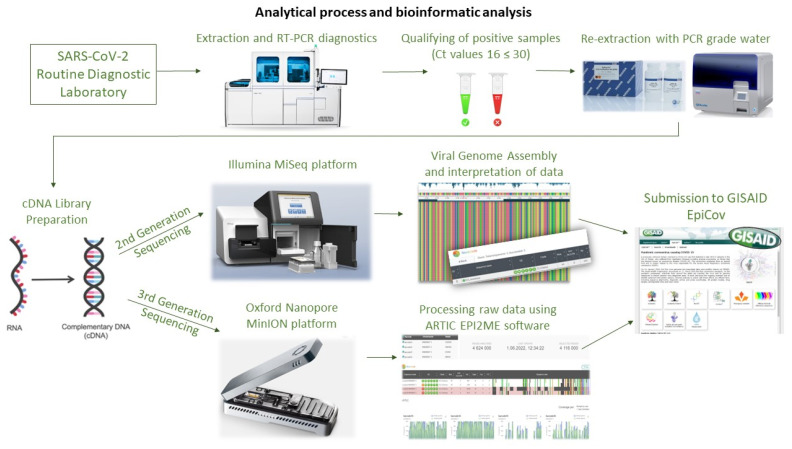
Analytical process and bioinformatics analysis. Cobas 6800: ”https://diagnostics.roche.com/us/en/products/instruments/cobas-6800-ins-2693.html (accessed on 14 July 2022)”. QIAcube Connect: ”https://biotech.ufl.edu/portfolio/qiagen-qiacube/ (accessed on 14 July 2022)”. MiSeq: “https://www.illumina.com/systems/sequencing-platforms/miseq/order-miseq.html (accessed on 22 July 2022)”. MinION Mk1B: “https://www.prnewswire.com/in/news-releases/oxford-nanopore-sequencers-have-left-uk-for-china-to-support-rapid-near-sample-coronavirus-sequencing-for-outbreak-surveillance-856474669.html (accessed on 14 July 2022)”.

The study aimed to determine the genetic variability of SARS-CoV-2 in samples collected from Polish military personnel performing duties in Poland and engaged in operations and missions abroad in Kosovo, Romania, Iraq, and France. The swab samples were collected by The Military Center of Preventive Medicine in Wrocław and tested at the Biological Threats Identification and Countermeasure Centre in Puławy. During the six-month study period (November 2021–May 2022), clinical samples collected from 1699 Polish soldiers were routinely tested for SARS-CoV-2 infections using PCR tests before and after the mission as part of routine active surveillance to prevent the introduction and spread of COVID-19 among military personnel. The schematic representation of the process is presented in Figure 9.

Initially, all samples were analysed using the Roche Cobas 6800^®^ system (Roche Diagnostics Corporation, Indianapolis, IN, USA) with the Roche SARS-CoV-2 test, which identifies two specific gene targets (*ORF1ab* and *E* gene). Subsequently, a total of 84 out of 223 SARS-CoV-2 positive samples with suitable virus titres (threshold cycle (Ct) values between 16 and 30) originating from soldiers aged between 20 and 52 years were selected for further steps of SARS-CoV-2 WGS (whole genome sequencing). RNA was extracted and sequenced using the Illumina MiSeq and Oxford Nanopore MinION platforms. The sequencing data from MiSeq were generated as part of multiplex runs of 81 SARS-CoV-2 positive isolates, while Oxford’s sequencing was performed with three SARS-CoV-2 positive isolates per flow cell in two runs. Since MiSeq and MinION are both highly reliable and widely used sequencing platforms, the selection of a particular platform was primarily driven by their availability and the feasibility of their use within the context of our surveillance program; thus, the choice was not based on any specific rationale related to the nature of the samples or the expected outcomes. Finally, analysed sequencing data have been uploaded to GISAID (Global Initiative on Sharing All Influenza Data), a global database of sequenced SARS-CoV-2 genomes for public access [32].

### 3.2. Sampling

Nasopharyngeal and oropharyngeal swabs were collected from military personnel in viral transport medium (VTM) and stored at low temperatures (2–8 °C) during transport, following the recommendations of the European Centre for Disease Prevention and Control (ECDC) and the World Health Organization (WHO), to preserve the diagnostic features of the samples [12]. The diagnostic procedures with the samples were carried out in a Biosafety Level 2 (BSL2) laboratory, following the principles of Good Laboratory Practice (GLP) and Standard Operating Procedures (SOPs) developed for the collection, deposition, and sharing of samples related to the sequencing of the SARS-CoV-2 genome.

### 3.3. Viral RNA Extraction and Quality Assessment

RNA was extracted automatically using the QIAamp Viral RNA Mini kit (Qiagen, Valencia, CA, USA) on the QIAcube apparatus (Qiagen, Valencia, CA, USA), or manually with the same kit. RNA was purified from 140 µL of each sample, eluted with 60 µL of PCR-grade water, and stored at −80 °C. The qualification criteria for isolated material for whole genome sequencing were based on microcapillary electrophoresis using the Agilent 4150 TapeStation System (Agilent Technologies, Waldbronn, Germany) and the viral load in the RNA solution. Ultimately, 84 samples met the quality criteria and were approved for sequencing and viral genome analysis. The same RNA extract was used for MiSeq and MinION sequencing library preparation.

### 3.4. Illumina Sequencing

RNA was extracted from 84 qualified viral samples and transcribed to cDNA using the LunaScript™ Reverse Transcription SuperMix kit (New England Biolabs, Hitchin, UK) following the manufacturer’s instructions. The cDNA, covering the 29.9 kb SARS-CoV-2 genome, was used to prepare Illumina-compatible sequencing libraries using the EasySeq™ RC-PCR SARS-CoV-2 WGS kit (NimaGen BV, Nijmegen, The Netherlands). Library construction was based on reverse complement polymerase chain reaction (RC-PCR), a technology that combines target amplification and indexing in a single reaction. The sequence-ready libraries were purified using an AmpliCleanTM beads solution and quantified using the QuantiFluor^®^ dsDNA System kit (Promega, Madison, WI, USA) on a Quantus™ Fluorometer (Promega, Madison, WI, USA) following the manufacturer’s manual. The libraries were analysed on TapeStation (Agilent Technologies, Santa Clara, CA, USA), with expected amplicon fragments ranging between 400 and 500 bp in size, before being sequenced on an Illumina next-generation sequencing (NGS) platform. In summary, the 9 pM library was spiked with 12.5 pM PhiX control (#FC-110-3001, Illumina) and sequenced on a MiSeq sequencer by Illumina with 150-bp paired-end reads, using the recommended MiSeq Reagent Kit v2 (300 cycles). The raw sequencing data were demultiplexed and extracted in fastq format.

### 3.5. Bioinformatic Analysis

The quality of the paired-end reads generated from Illumina sequencing was checked using FastQC and trimmed. The final reads were mapped against the reference genome sequence (NC_045512.2, MN908947.3) using the Bowtie2 tool and manually verified using Unipro UGENE v.41 [33]. All consensus sequences have been deposited in GISAID (accession numbers in Supplementary Table S1).

### 3.6. MinION Sequencing

Three SARS-CoV-2 positive samples were selected for nanopore sequencing using the Oxford Nanopore Technologies (ONT) MinION platform. The sequencing procedure involved the application of the manufacturer’s PCR tiling method specific to the SARS-CoV-2 virus, incorporating rapid barcoding (SQK-RBK110.96) and Midnight RT PCR Expansion (EXP-MRT001) protocols. The samples exhibited Ct values ranging from 21 to 25, indicating varying viral loads. For PCR amplification, the manufacturer’s instructions were followed, using 1200 base pairs amplicon Midnight primers. Subsequently, sequencing libraries were generated using the rapid barcoding kit. Pooled barcoded libraries were purified using AMPure XP beads (Beckman Coulter Diagnostics, Brea, CA, USA) in a 1:1 ratio and eluted in 15 µL elution buffer. Following priming, 800 ng of the libraries were loaded onto an R9.4.1 flow cell (FLO-MIN106D) and subjected to 72 h of sequencing on a MinION (Mk1B) device (ONT, Oxford, UK).

The MinKNOW software (Oxford Nanopore Technology, version 21.11.8) was employed for data collection, basecalling, and demultiplexing, resulting in a total of 3.39 Gb of raw sequencing data. The obtained genome sequences encompassed approximately 29.9 kb nucleotides and exhibited a minimum coverage depth of 400. The first ONT’s sequencing run, which included the sample EPI_ISL_11268036, was conducted in March 2022, utilizing the ARTIC EPI2ME v3.4.1 SARS-CoV-2 pipeline (FastQC plus Artic plus Nextclade v2022.03.08). Subsequently, reads from the second run, involving samples EPI_ISL_13159732 and EPI_ISL_13159733, were exported as FASTQ files and processed using the same ARTIC pipeline (FastQC plus Artic plus Nextclade v2022.04.26-13521) in May 2022. All consensus sequences derived from the nanopore sequencing runs have been deposited in the Global Initiative on Sharing All Influenza Data (GISAID), with corresponding accession numbers provided in Supplementary Table S1.

### 3.7. Ethical Issues

The research detailed in this manuscript was carried out as part of routine examinations for SARS-CoV-2 among military personnel. This was done in strict accordance with the document MON/12-03/23 and the Guidelines for carrying out research on sequencing the genome of the SARS-CoV-2 virus in the Ministry of National Defence for the purposes of epidemiological surveillance, issued in September 2022 by the Department of the Military Health Service of the Ministry of National Defence. Additionally, the study adhered to order No. 51/MON by the Ministry of National Defence dated 30 December 2019. This investigation was conducted following stringent anonymization guidelines and is part of the broader epidemiological surveillance efforts. Therefore, no additional ethical approval was necessary for its publication.

## 4. Conclusions

The findings of this study provide insights into the genetic variability of SARS-CoV-2 among members of the Polish Armed Forces (PAF) deployed on missions abroad. We confirmed that all SARS-CoV-2 genetic variants detected in PAF members were already present in Poland before their return from missions, suggesting that either there was a lack of transmission of these variants to the national environment, or any transmissions that did occur did not significantly change the SARS-CoV-2 repertoire in Poland.

The implementation of the Military Sanitary Inspection recommendations, including pre-departure and early post-mission screening testing and isolation, played a crucial role in preventing both the export and import of SARS-CoV-2 variants among military personnel. These measures safeguarded the readiness and ability of the PAF to conduct missions and operations effectively. The low number of infections among returning military personnel further highlights the efficacy of the infection mitigation measures implemented during these missions.

However, to gain a more comprehensive understanding of infection dynamics during missions, it would be beneficial to conduct routine PCR screening tests and determine specific antibody levels. This additional information would provide valuable insights into the detailed dynamics of infections among military personnel.

Overall, this study provides valuable insights for public health authorities and military organizations in developing strategies to mitigate the spread of infectious diseases among deployed military personnel and safeguarding global health security.

## Data Availability

The SARS-CoV-2 genome sequences included in the study were submitted to the GISAID (Global Initiative on Sharing All Influenza Data) repository (www.gisaid.org accessed on 18 August 2022). Their access IDs are given in Tables S1 and S3.

## Supplementary Materials

The following supporting information can be downloaded at: https://www.mdpi.com/article/10.3390/ijms241914851/s1.
