# Supplementary material for: Surveillance of SARS-CoV-2 Genetic Variants in the Polish Armed Forces Using Whole Genome Sequencing Analysis
Source: Int J Mol Sci. 2023 Oct 3;24(19):14851. doi: 10.3390/ijms241914851 (PMC10573488; doi:10.3390/ijms241914851)
Supplement: Supplementary file 1 [file ijms-24-14851-s001.zip › ijms-2555253-supplementary/figures & supplements/Supplementary Table S1.pdf]

GISAID	Date of sample collection (day.month .yr)	Patient age (yr)	Vaccination	Nextstrain clades	Pangolin lineage	Coverage x	GC content (%)	Location	Deployme nt
EPI_ISL_13156671	09.11.2021	38	1A, 2A	Delta (21J)	B.1.617.2	905x	38,12%	Poland	pre-
EPI_ISL_13156590	27.11.2021	35	1C, 2C	Delta (21J)	AY.4.4	2079x	38,20%	Poland	pre-
EPI_ISL_11881929	24.01.2022	33	1J	Omicron (21K)	BA.1	1708x	38,20%	Poland	pre-
EPI_ISL_11881930	27.01.2022	34	1A, 2A, 3C	Omicron (21K)	BA.1	1105x	38,14%	Poland	pre-
EPI_ISL_11881931	26.01.2022	38	1V, 2V, 3C	Omicron (21K)	BA.1	1521x	38,20%	Poland	pre-
EPI_ISL_9694159	30.01.2022	25	1A, 2A	Omicron (21K)	BA.1.1	1332x	38,17%	Kosovo	post-
EPI_ISL_9694160	30.01.2022	35	1A, 2A	Omicron (21K)	BA.1.1	2233x	38,12%	Kosovo	post-
EPI_ISL_9694161	30.01.2022	31	1A, 2A	Omicron (21K)	BA.1	1362x	38,22%	Kosovo	post-
EPI_ISL_11881932	30.01.2022	31	1A, 2A	Omicron (21K)	BA.1.1	1915x	38,10%	Kosovo	post-
EPI_ISL_9694162	30.01.2022	35	1A, 2A	Omicron (21K)	BA.1.1	2886x	38,15%	Kosovo	post-
EPI_ISL_9694163	30.01.2022	37	1A, 2A	Omicron (21K)	BA.1.1	719x	38,13%	Kosovo	post-
EPI_ISL_11881933	30.01.2022	29	1A, 2A	Omicron (21K)	BA.1.1	5704x	38,23%	Kosovo	post-
EPI_ISL_9694164	30.01.2022	41	1C, 2C	Omicron (21K)	BA.1.1	1694x	38,14%	Kosovo	post-
EPI_ISL_9694165	30.01.2022	28	1A, 2A	Omicron (21K)	BA.1.1	1768x	38,20%	Kosovo	post-
EPI_ISL_11881934	30.01.2022	34	1A, 2A	Omicron (21K)	BA.1.1	1934x	38,10%	Kosovo	post-
EPI_ISL_9963991	30.01.2022	33	1A, 2A	Omicron (21K)	BA.1.1	2034x	38,20%	Kosovo	post-
EPI_ISL_11881935	30.01.2022	35	1C, 2C	Omicron (21K)	BA.1.1	1548x	38,22%	Kosovo	post-
EPI_ISL_9963992	30.01.2022	32	1A, 2A	Omicron (21K)	BA.1.1	2577x	38,16%	Kosovo	post-
EPI_ISL_11881936	30.01.2022	24	1A, 2A	Omicron (21K)	BA.1.1	2106x	38,12%	Kosovo	post-
EPI_ISL_11881937	30.01.2022	34	1M, 2M	Omicron (21K)	BA.1.1	1832x	38,20%	Kosovo	post-
EPI_ISL_11881938	30.01.2022	26	1A, 2A	Omicron (21K)	BA.1.1	2369x	38,15%	Kosovo	post-
EPI_ISL_12253975	25.01.2022	24	1A, 2A	Omicron (21K)	BA.1.1.1	1227x	38,17%	Poland	HO
EPI_ISL_9963993	25.01.2022	20	1J	Omicron (21K)	BA.1	2561x	38,14%	Poland	HO
EPI_ISL_12253976	25.01.2022	22	1J	Omicron (21K)	BA.1	2102x	38,15%	Poland	HO
EPI_ISL_9963994	01.02.2022	36	1A, 2A, 3C	Omicron (21K)	BA.1	1256x	38,14%	Poland	pre-
EPI_ISL_12253977	24.01.2022	29	1A, 2A, 3C	Omicron (21K)	BA.1	1748x	38,13%	Poland	HO

EPI_ISL_12253978	24.01.2022	40	1M, 2M, 3C*	Omicron (21K)	BA.1	1420x	38,21%	Poland	HO
EPI_ISL_9963995	24.01.2022	46	1M, 2M, 3C	Omicron (21K)	BA.1	1494x	38,12%	Poland	HO
EPI_ISL_12253979	24.01.2022	30	1M, 2M, 3C	Omicron (21K)	BA.1.1	1485x	38,12%	Poland	HO
EPI_ISL_9963996	30.01.2022	40	1A, 2A, 3C	Omicron (21K)	BA.1	1606x	38,16%	Poland	HO
EPI_ISL_9963997	30.01.2022	27	1A, 2A	Omicron (21K)	BA.1.1	1545x	38,17%	Kosovo	post-
EPI_ISL_10008058	30.01.2022	52	1J	Omicron (21K)	BA.1.1	1663x	38,18%	Poland	pre-
EPI_ISL_10008059	30.01.2022	35	1A, 2A	Omicron (21K)	BA.1.1	2350x	38,22%	Kosovo	post-
EPI_ISL_10008060	30.01.2022	36	1A, 2A	Omicron (21K)	BA.1.1	1525x	38,21%	Kosovo	post-
EPI_ISL_10008061	30.01.2022	33	1A, 2A	Omicron (21K)	BA.1.1	2179x	38,22%	Kosovo	post-
EPI_ISL_10008062	30.01.2022	30	1A, 2A	Omicron (21K)	BA.1.1	2245x	38,15%	Kosovo	post-
EPI_ISL_10008064	30.01.2022	31	1A, 2A	Omicron (21K)	BA.1.1	1574x	38,14%	Kosovo	post-
EPI_ISL_12253980	30.01.2022	24	1A, 2A	Omicron (21K)	BA.1.1	1672x	38,17%	Kosovo	post-
EPI_ISL_10008065	30.01.2022	32	1A, 2A	Omicron (21K)	BA.1.1	1604x	38,15%	Kosovo	post-
EPI_ISL_10008066	30.01.2022	33	1A, 2A	Omicron (21K)	BA.1.1	3072x	38,14%	Kosovo	post-
EPI_ISL_10008067	30.01.2022	38	1A, 2A	Omicron (21K)	BA.1.1	2203x	38,15%	Kosovo	post-
EPI_ISL_12253981	30.01.2022	38	1A, 2A	Omicron (21K)	BA.1.1	2916x	38,16%	Kosovo	post-
EPI_ISL_10008068	30.01.2022	38	1A, 2A	Omicron (21K)	BA.1.1	1305x	38,12%	Kosovo	post-
EPI_ISL_10008069	25.01.2022	24	1C, 2C	Omicron (21K)	BA.1.1.1	1297x	38,22%	Poland	HO
EPI_ISL_9694166	25.01.2022	20	1J, 2C	Omicron (21K)	BA.1	1796x	38,16%	Poland	HO
EPI_ISL_12253982	25.01.2022	21	1C, 2C	Omicron (21K)	BA.1	1656x	38,14%	Poland	pre-
EPI_ISL_9694167	25.01.2022	20	1A, 2A	Omicron (21K)	BA.1.1	1317x	38,20%	Poland	HO
EPI_ISL_12253983	25.01.2022	21	1A, 2A, 3C	Omicron (21K)	BA.1.1	1065x	38,17%	Poland	HO
EPI_ISL_12253984	24.01.2022	45	1J	Omicron (21K)	BA.1	1595x	38,16%	Poland	HO
EPI_ISL_9694168	01.02.2022	39	1M, 2C	Omicron (21K)	BA.1.1.1	1697x	38,16%	Poland	pre-
EPI_ISL_9694169	01.02.2022	36	1C, 2C, 3C*	Omicron (21K)	BA.1.1.1	1331x	38,12%	Poland	pre-
EPI_ISL_12253985	01.02.2022	30	1C	Omicron (21K)	BA.1.1	1678x	38,14%	Poland	pre-
EPI_ISL_12253986	01.02.2022	41	1A, 2A, 3C	Omicron (21K)	BA.1.15.1	887x	38,22%	Poland	pre-
EPI_ISL_12253987	01.02.2022	45	1A, 2A, 3C	Omicron (21K)	BA.1	1807x	38,10%	Poland	pre-
EPI_ISL_11313684	04.02.2022	43	1A, 2A	Omicron (21K)	BA.1.1	2006x	38,14%	Romania	post-
EPI_ISL_11313685	04.02.2022	28	1A, 2A	Omicron (21K)	BA.1	1589x	38,16%	Romania	post-
EPI_ISL_11313686	04.02.2022	27	1A, 2A	Omicron (21K)	BA.1.1	3023x	38,15%	Kosovo	post-

EPI_ISL_11313687	04.02.2022	29	1A, 2A	Omicron (21K)	BA.1.1	1815x	38,10%	Kosovo	post-
EPI_ISL_11313688	04.02.2022	31	1A, 2A	Omicron (21K)	BA.1.1	2313x	38,15%	Kosovo	post-
EPI_ISL_11313689	08.02.2022	36	1A, 2A, 3C	Omicron (21L)	BA.2.9	3956x	38,17%	Poland	pre-
EPI_ISL_11313690	08.02.2022	26	1A, 2A, 3C	Omicron (21L)	BA.2	1827x	38,10%	Poland	pre-
EPI_ISL_11313691	11.02.2022	29	1A, 2A, 3C	Omicron (21K)	BA.1	1101x	38,13%	Romania	post-
EPI_ISL_11313692	15.02.2022	25	1A, 2A, 3C	Omicron (21L)	BA.2.9	1942x	38,11%	Poland	pre-
EPI_ISL_11313693	15.02.2022	25	1C, 2C, 3C	Omicron (21K)	BA.1	3041x	38,14%	Poland	pre-
EPI_ISL_11313694	15.02.2022	30	1C, 2C	Omicron (21L)	BA.2	2230x	38,15%	Poland	pre-
EPI_ISL_11313695	15.02.2022	33	1A, 2A, 3C	Omicron (21K)	BA.1.17.2	876x	38,16%	Poland	pre-
EPI_ISL_11313696	15.02.2022	45	1C, 2C, 3C	Omicron (21K)	BA.1	1430x	38,14%	Poland	pre-
EPI_ISL_11313697	17.02.2022	38	1A, 2A	Omicron (21L)	BA.2	1362x	38,12%	Romania	post-
EPI_ISL_11313698	17.02.2022	42	1A, 2A	Omicron (21K)	BA.1.1.13	142x	38,13%	Romania	post-
EPI_ISL_11349025	17.02.2022	25	1A, 2A	Omicron (21L)	BA.2	1220x	38,22%	Romania	post-
EPI_ISL_11349026	04.02.2022	31	1A, 2A	Omicron (21K)	BA.1.1	1591x	38,16%	Kosovo	post-
EPI_ISL_11349027	04.02.2022	32	1J	Omicron (21K)	BA.1.1	1700x	38,17%	Kosovo	post-
EPI_ISL_11349028	04.02.2022	24	1A, 2A	Omicron (21K)	BA.1.1	1934x	38,11%	Kosovo	post-
EPI_ISL_11349029	04.02.2022	41	1A, 2A	Omicron (21K)	BA.1.1	2154x	38,15%	Kosovo	post-
EPI_ISL_11268036	04.02.2022	42	1A, 2A	Omicron (21K)	BA.1.1	400x	37,94%	Kosovo	post-
EPI_ISL_11349030	04.02.2022	40	1A, 2A	Omicron (21K)	BA.1.1	1770x	38,17%	Kosovo	post-
EPI_ISL_11349031	04.02.2022	34	1A, 2A	Omicron (21K)	BA.1.1	1106x	38,12%	Kosovo	post-
EPI_ISL_11349032	04.02.2022	38	1A, 2A	Omicron (21K)	BA.1.1	1063x	38,13%	Kosovo	post-
EPI_ISL_11349033	04.02.2022	31	1A, 2A	Omicron (21K)	BA.1.1	581x	38,14%	Kosovo	post-
EPI_ISL_11349034	04.02.2022	39	1A, 2A	Omicron (21K)	BA.1.1	2224x	38,16%	Kosovo	post-
EPI_ISL_11349035	04.02.2022	25	1A, 2A	Omicron (21K)	BA.1.1	2818x	38,22%	Kosovo	post-
EPI_ISL_11349036	04.02.2022	23	1M, 2M	Omicron (21K)	BA.1.1	2394x	38,15%	Kosovo	post-
EPI_ISL_13159732	20.05.2022	26	1A, 2A, 3C	Omicron (21L)	BA.2.9	400x	38,37%	France	post-
EPI_ISL_13159733	20.05.2022	22	1C, 2C, 3C	Omicron (21L)	BA.2.56	400x	38,48%	France	post-

Abbreviations: GISAID, Global Initiative on Sharing All Influenza Data; GC content, percentage of guanine and cytosine in the nucleotide sequence; yr, year; 1, 1st dose of vaccine; 2, 2nd dose of vaccine; 3, 3rd dose of vaccine; A, AstraZeneca COVID-19 Vaccine; C, mRNA Comirnaty COVID-19 Vaccine; J, viral vector-Janssen COVID-19 Vaccine; M, mRNA Moderna COVID-19 Vaccine; V, Vaxzevria, COVID-19 Vaccine (previously COVID-19 Vaccine AstraZeneca); vaccination status marked with asterix * - less than 14 days from the vaccination with the last dose to diagnosed infection; pre – sample collected before deployment; post – sample collected after deployment; HO – sample collected for diagnostics of home outbreak
