# Supplementary figures and images for: Surveillance of SARS-CoV-2 Genetic Variants in the Polish Armed Forces Using Whole Genome Sequencing Analysis
Source: Int J Mol Sci. 2023 Oct 3;24(19):14851. doi: 10.3390/ijms241914851 (PMC10573488; doi:10.3390/ijms241914851)

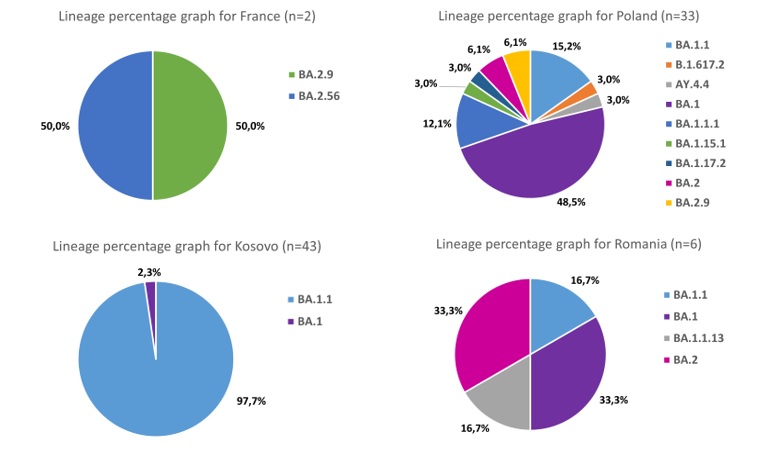

Supplement: Supplementary file 1 [file ijms-24-14851-s001.zip › ijms-2555253-supplementary/figures & supplements/Figure 1.jpg]

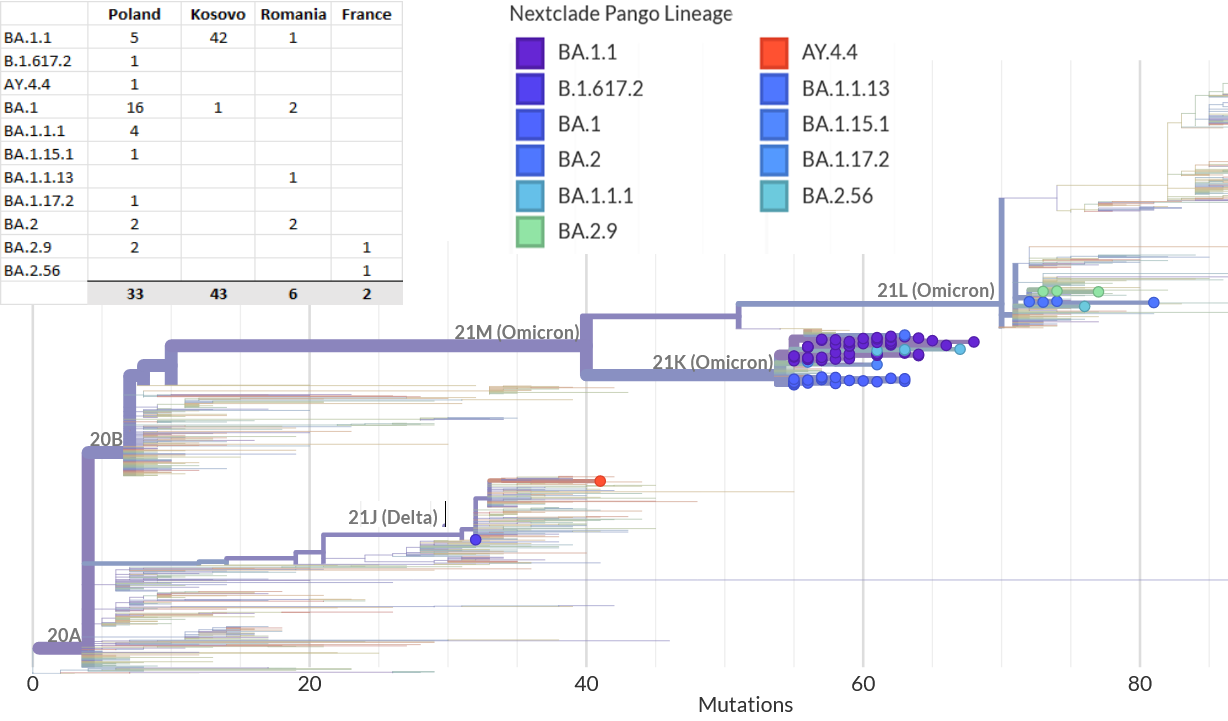

Supplement: Supplementary file 1 [file ijms-24-14851-s001.zip › ijms-2555253-supplementary/figures & supplements/Figure 2.bmp]

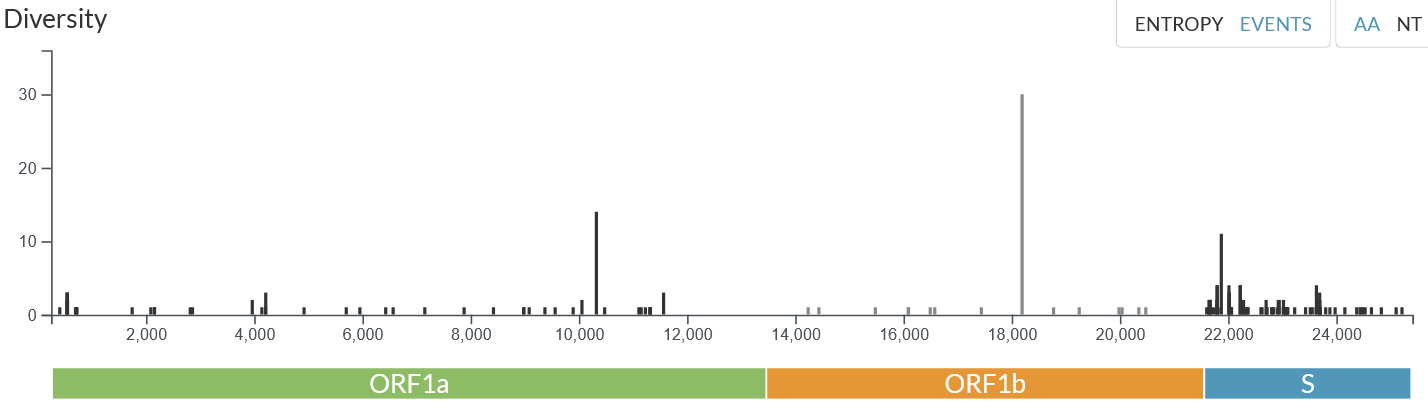

Supplement: Supplementary file 1 [file ijms-24-14851-s001.zip › ijms-2555253-supplementary/figures & supplements/Figure 3A.bmp]

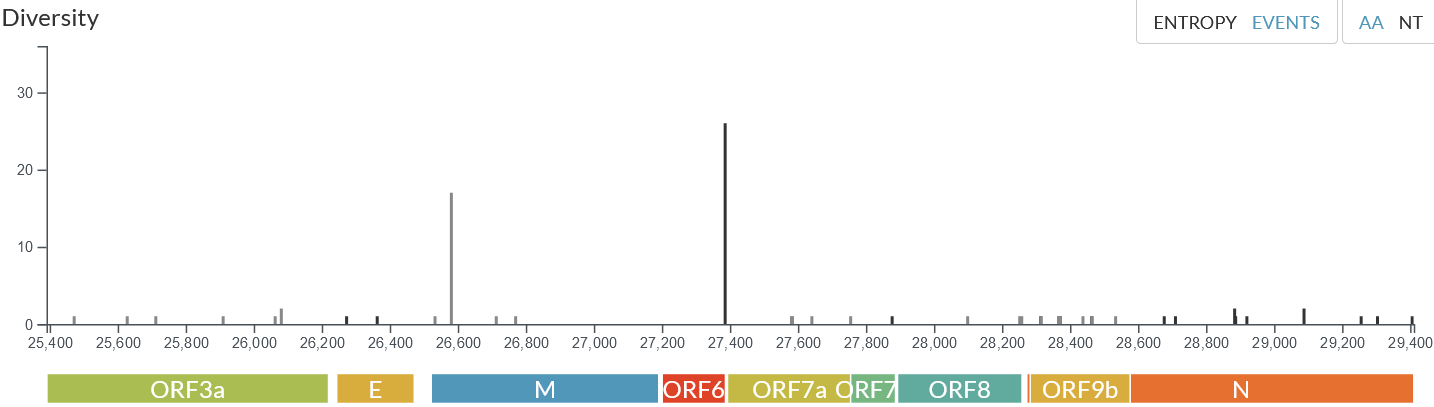

Supplement: Supplementary file 1 [file ijms-24-14851-s001.zip › ijms-2555253-supplementary/figures & supplements/Figure 3B.bmp]

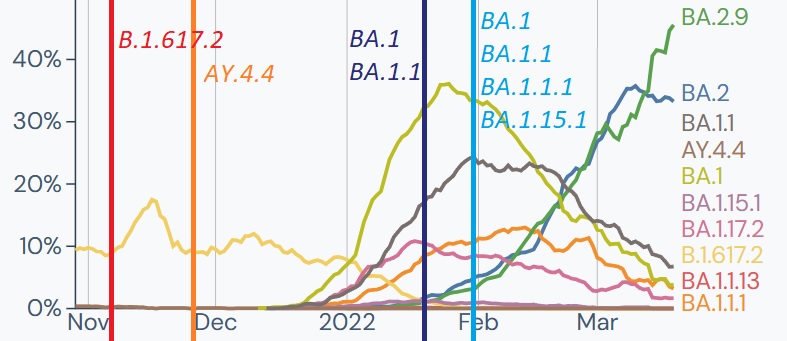

Supplement: Supplementary file 1 [file ijms-24-14851-s001.zip › ijms-2555253-supplementary/figures & supplements/Figure 4.jpg]

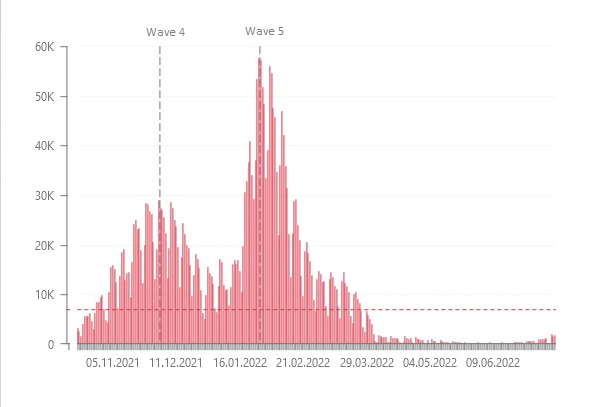

Supplement: Supplementary file 1 [file ijms-24-14851-s001.zip › ijms-2555253-supplementary/figures & supplements/Figure 5.tif]

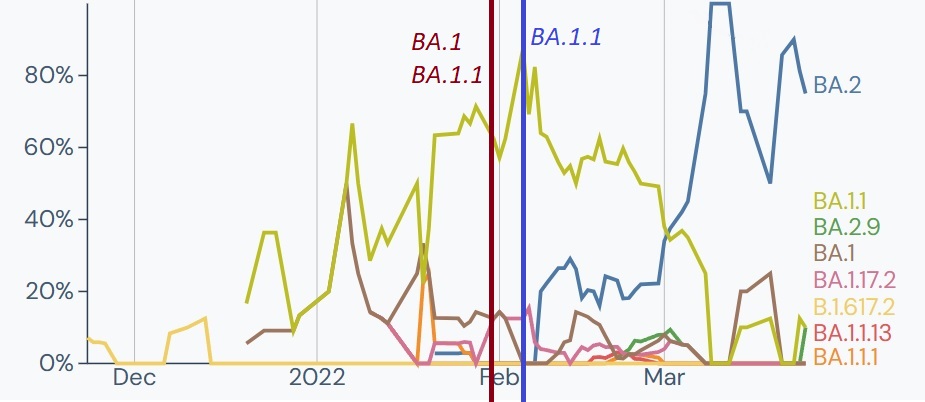

Supplement: Supplementary file 1 [file ijms-24-14851-s001.zip › ijms-2555253-supplementary/figures & supplements/Figure 6.jpg]

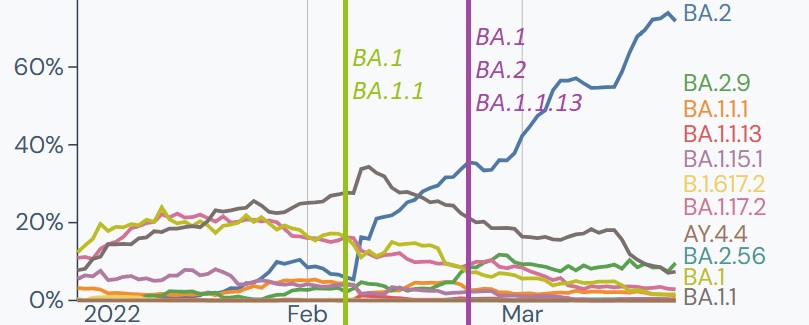

Supplement: Supplementary file 1 [file ijms-24-14851-s001.zip › ijms-2555253-supplementary/figures & supplements/Figure 7.jpg]

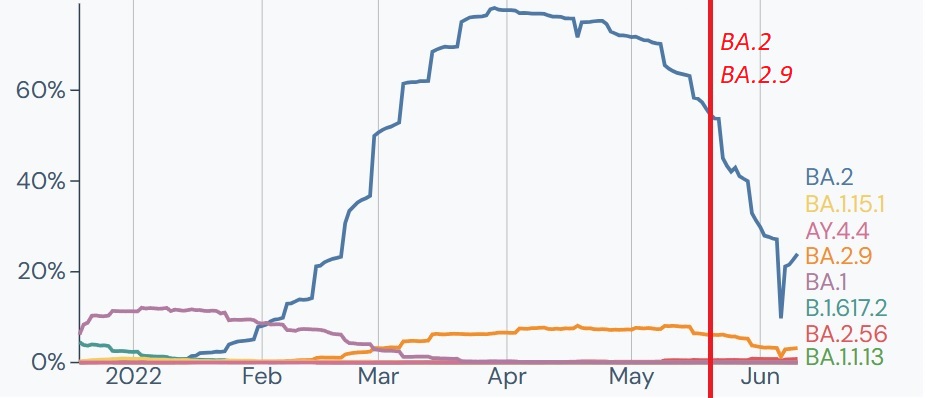

Supplement: Supplementary file 1 [file ijms-24-14851-s001.zip › ijms-2555253-supplementary/figures & supplements/Figure 8.jpg]

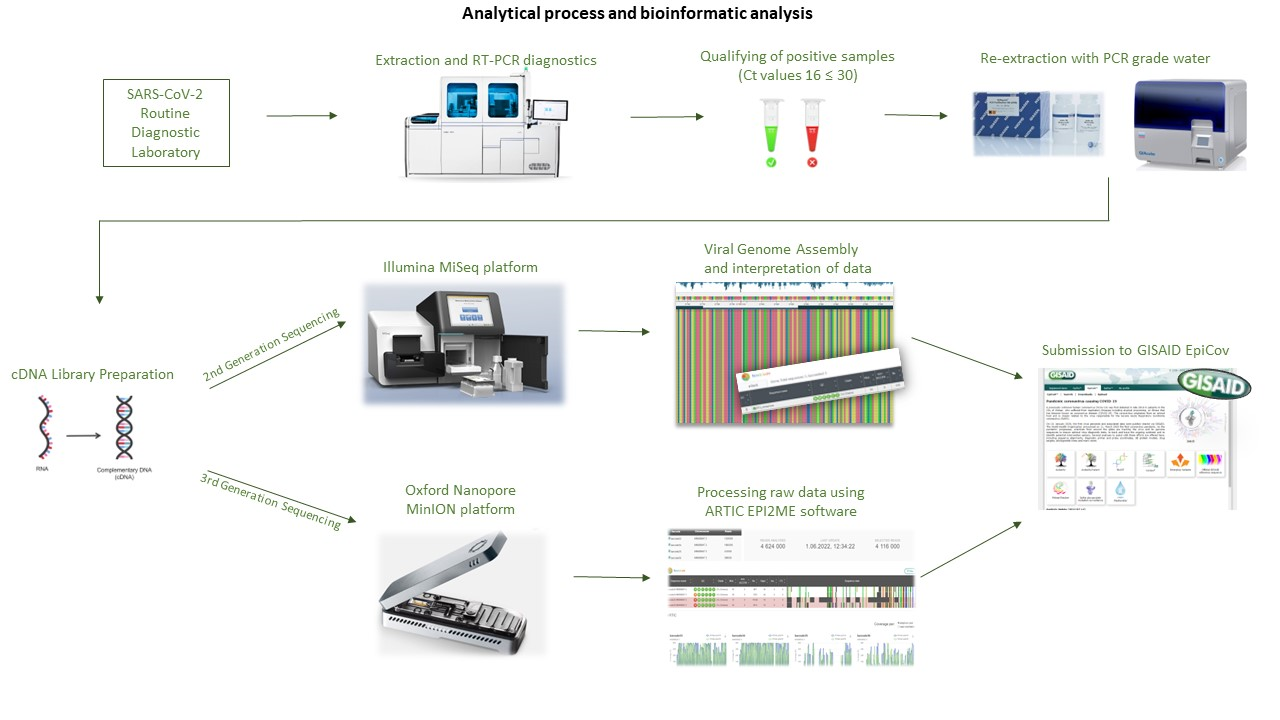

Supplement: Supplementary file 1 [file ijms-24-14851-s001.zip › ijms-2555253-supplementary/figures & supplements/Figure 9.tif]

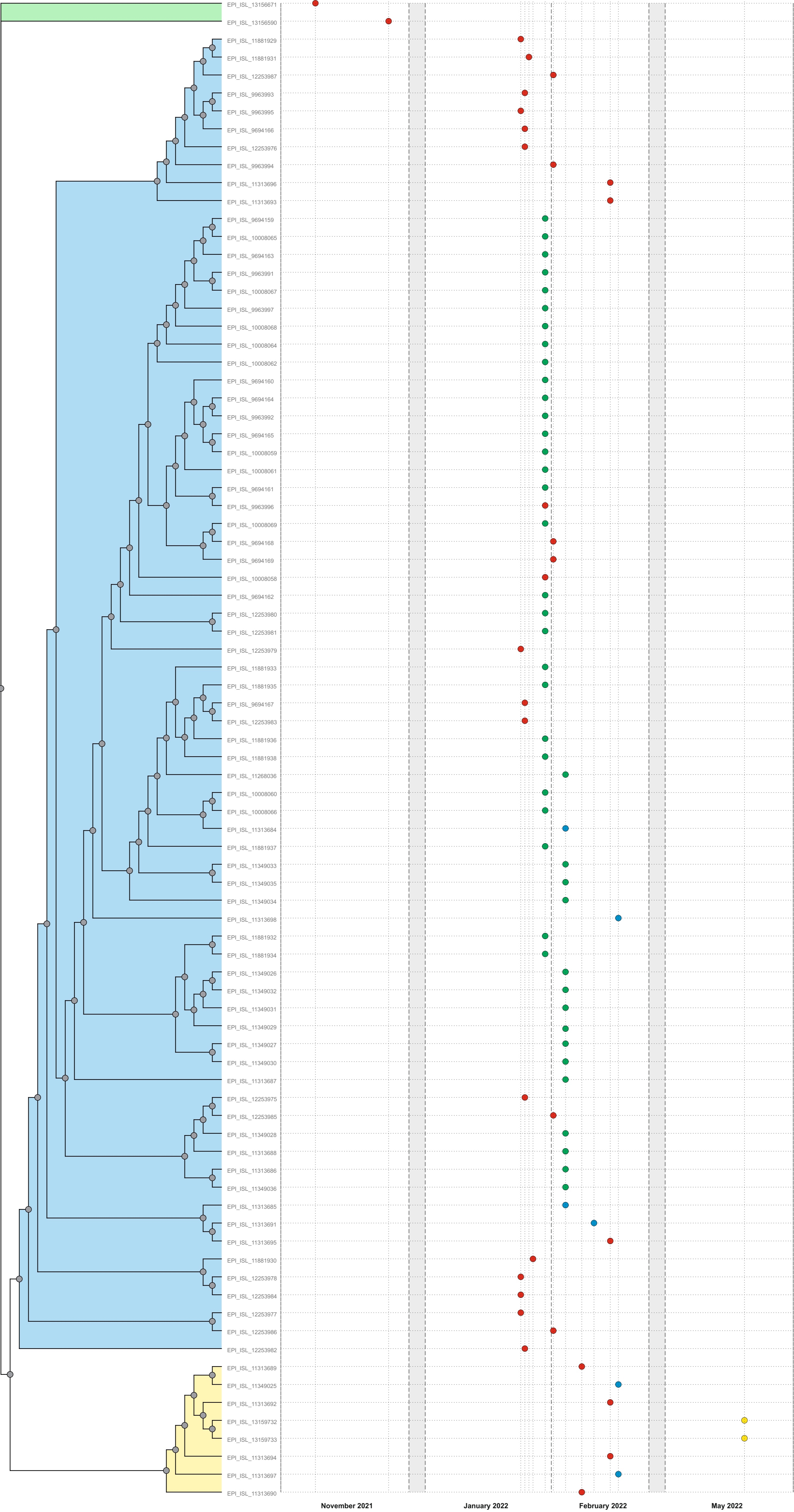

Supplement: Supplementary file 1 [file ijms-24-14851-s001.zip › ijms-2555253-supplementary/figures & supplements/Supplementary Figure S2.pdf]

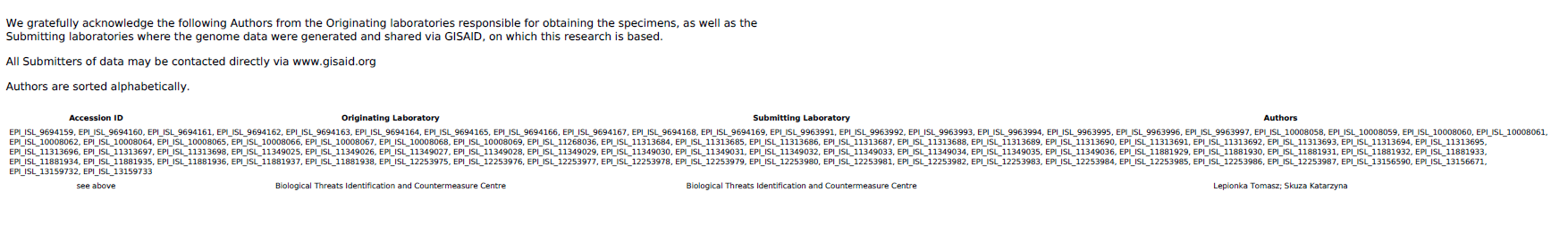

Supplement: Supplementary file 1 [file ijms-24-14851-s001.zip › ijms-2555253-supplementary/figures & supplements/Supplementary_Table S3.tif]
